# The Chinese version of the pediatric quality of life inventory™ (PedsQL™) healthcare satisfaction generic module (version 3.0): psychometric evaluation

**DOI:** 10.1186/1477-7525-11-113

**Published:** 2013-07-05

**Authors:** Jialing Li, Lianxiong Yuan, Yu Wu, Yunzhu Luan, Yuantao Hao

**Affiliations:** 1 Department of Medical Statistics and Epidemiology, School of Public Health, Sun Yat-sen University, Guangzhou 510080, the People’s Republic of China; 2 School of Foreign Language, Southern Medical University, Guangzhou 510515, the People’s Republic of China

**Keywords:** Pediatric, Healthcare satisfaction, Reliability, Validity

## Abstract

**Background:**

Healthcare satisfaction is an important indicator in quality of healthcare evaluations. Instruments with psychometric evaluation for pediatric healthcare satisfaction in China have been rarely studied. The PedsQL™ 3.0 Healthcare Satisfaction Generic Module was a widely used instrument designed to measure parents’ satisfaction of the healthcare for their children with chronic diseases. This study aimed to evaluate the psychometric properties of the Chinese version of the PedsQL™ 3.0 Healthcare Satisfaction Generic Module.

**Methods:**

We used the standard procedure of cross-culture adaptation to develop the Chinese version of the PedsQL™ 3.0 Healthcare Satisfaction Generic Module. We administered the scale to 354 parents with children experiencing chronic diseases from 5 third-grade class-A hospitals in Guangzhou by the convenience sampling method. The psychometric properties of the scale were evaluated.

**Results:**

The response rate was 94.4%. All the subscales reached the minimum reliability standard of 0.70 for Cronbach’s alpha coefficient and the recommended test-retest reliability standard of 0.80 for intra-class correlation coefficients (*ICC*s). There were higher correlation coefficients between items and their hypothesized subscales than those with other subscales. Confirmatory factor analysis showed that the scale had adequate construct validity with the main indexes of goodness of fit *CFI* and *RMSEA* as 0.99 and 0.078, respectively.

**Conclusions:**

The results demonstrate that the Chinese version of the PedsQL™ 3.0 Healthcare Satisfaction Generic Module is a feasible instrument with adequate psychometric properties.

## Background

With the changes of human disease spectrum, the prevalence of pediatric chronic diseases has increased significantly in recent decades [1,2]. There are between 8% and 20% of the global pediatric population living with chronic diseases [2]. The long hospitalization and treatment course of chronic diseases lead to two significant changes for pediatric patients and their families. Firstly, long treatment courses and hospitalizations results in increased time spent in bed leading to lack of playtime, lack of communication with friends, and greater burdens for their families. Such factors results in a series of physical and emotional symptoms, cognitive problems, and social adaption problems, ultimately greatly impacting family interactions. Secondly, traditional evaluation indicators such as cure rate and survival rate are not comprehensive enough for patients with chronic diseases. Instead of the cure rate and survival rate, people prefer to be concerned with the experience of the patients and their families in the long disease duration such as the satisfaction of the healthcare. Healthcare quality is a very importance influencing factor on the quality of life for pediatric patients and their families. One of the effective ways to assess the healthcare quality is the healthcare satisfaction assessments of patients/their families. Patient/parent satisfaction has been identified as one of the three major categories of criteria for the evaluation of health care systems [3]. Patients tend to adhere to the therapeutic regimens and seldom change the doctors when they are satisfied with the healthcare team [3,4]. Healthcare satisfaction assessments also may be helpful for healthcare professionals to improve their quality of care [5].

Pascoe stated that there were no scientific definitions and adequate psychological models in previous studies of patient satisfaction. He defined patient satisfaction as patient response and evaluation of the healthcare environment, process and outcome comparing with their expectation [6]. As healthcare satisfaction is an important indicator in the evaluation of the quality of healthcare, a well-designed questionnaire for healthcare satisfaction assessment is of increasing importance. However, a literature search through Pub Med, Web of Knowledge, and Medline for patient/parent satisfaction in pediatric area revealed few well designed and psychometrically evaluated instruments with Chinese versions.

Since the pediatric patients are too young to expect the quality of healthcare and understand the explanation for the diseases, healthcare staffs often discuss the details with the parents of patients. So the parents know more about the overall condition than the patients themselves, and to some extent, the parental satisfaction may represent the whole family’s satisfaction including the pediatric patient [7-10]. In order to improve the assessment of pediatric healthcare satisfaction for the parents in China, we decided to introduce and establish the Chinese version of the Pediatric Quality of Life Inventory (PedsQL™) 3.0 Healthcare Satisfaction Generic Module. The PedsQL™ Measurement Model, originally developed by James W. Varni et al. in 1999, is a promising Health-related quality of life instrument designed for pediatric patients aged 2–18 years [11]. With progressive application and modification, it has developed into a questionnaire series including the General Core Scale and disease-special Module, Family Impaction Module, and Healthcare Satisfaction Module. All questionnaires were shown to be reliable and valid [12]. The PedsQL™ has been translated into many languages and applied widely in different countries [13-17]. Additionally, some modules of the PedsQL™ Measurement Model were translated into Chinese and were evaluated that they had adequate psychometric properties [17-19]. The Healthcare Satisfaction Generic Module was specially designed to evaluate parent satisfaction with the healthcare for their children with chronic diseases. However, the scale has not been translated into Chinese.

This study aimed to translate the PedsQL™ Healthcare Satisfaction Generic Module from English to Chinese and undertake a psychometrical test to determine whether it was suitable for assessing the parents’ satisfaction with the healthcare for their children with chronic diseases in China.

## Methods

### Subjects

Parents of pediatric patients aged 2 to 18 years were eligible for the study if their children were diagnosed with chronic diseases such as hematologic diseases/tumor, cardiovascular diseases, nephritic syndrome, asthma and diabetes conforming to the national diagnostic standards of China. The parents were enrolled if their children were hospitalized for at least 3 days or were outpatients that had follow-ups for more than 3 months. All the participants were approached with permission from their doctors. Parents were excluded if they were illiterate or reluctant to participate, or if their children were reported to be mentally retarded (Some items in the scale are not suitable for the children with mental retarded).

This study was approved by the Ethics Committee of School of Public Health, Sun Yat-sen University and obtained informed consent from all the subjects.

### Data collection

Four undergraduate students majoring in Preventive Medicine or Medical English were trained as interviewers by the project manager before the formal start of investigation. All the subjects were recruited from 5 third-grade class-A hospitals in Guangzhou, China between April 2012 and July 2012 by convenience sampling method. Third-grade class-A hospitals are the top-class hospitals in China which provide high-level medical services and implement high medical education and research tasks. The parents completed the questionnaires independently during the pediatric patients’ hospitalization or outpatient department visit, and the interviewers were available to answer questions during the investigation. There were “start time” and “finish time” set up in the beginning and ending of the questionnaire for the collection of completion time. The questionnaires were then collected and checked for any missing data or logical mistakes by the interviewers. A total of 43 compliable parents of the hospitalized patients were asked to fill out the PedsQL™ 3.0 Healthcare Satisfaction Generic Module repeatedly one week after the first interview in order to evaluate the test-retest reliability of the scale.

### Measures

#### PedsQL™ 3.0 healthcare satisfaction generic module

The Chinese version of PedsQL™ 3.0 Healthcare Satisfaction Generic Module was used in this study. The scale was developed as a parent-reported instrument to measure the parents’ satisfaction with the healthcare for their children with chronic diseases. This 24-item scale encompasses six subscales: Information (5 items), Inclusion of Family (4 items), Communication (5 items), Technical Skills (3 items), Emotional Needs (4 items), and Overall Satisfaction (3 items). The questionnaire asks about how happy the parents are with the care that their children and family have received at the hospital from the staff. A 5-point Likert responses scale is utilized for each item (0 = never, 1 = sometimes, 2 = often, 3 = almost always, 4 = always), and a *not applicable* option is added for subjects if the item is not suitable for them (When scoring, “*not applicable*” would be regarded as missing value according to the scoring manual). Items are transformed to a 0–100 scale linearly (0 = 0, 1 = 25, 2 = 50, 3 = 75, 4 =100) with higher scores indicating higher satisfaction. The subscale scores are computed as the sum of the items divided by the number of items answered. If more than 50% of the items in a subscale are missing, the subscale scores would not be computed [20].

#### PedsQL™ family information form

The PedsQL™ Family Information Form, which was also developed by James W. Varni et al. [21], has been cross-culturally translated into Chinese. It is completed by parents, contains the socio-demographic information including the child’s age, gender, disease duration, the parents’ marital status, education, occupation, family income, and payment method for the child’s medical care.

#### Cross-cultural adaptation

The project manager had been authorized to develop the Chinese version (Putonghua) of PedsQL™. The Chinese version of the PedsQL™ 3.0 Healthcare Satisfaction Generic Module was developed following the linguistic validation of the PedsQL, which consisted of 4 steps: forward translation, backward translation, preliminary test, and field test [22]. The aim of the linguistic validation of the PedsQL is to produce a Chinese version which is conceptually equivalent to the original version, as well as clear and easy to understand.

The forward translation from English to Chinese was implemented by a pediatrician and a medical English teacher independently, both of whom had a good command of English and Chinese. The two drafts were then discussed by a multidisciplinary team which consisted of translators, a health services researcher, and a project manager in order to produce a combined Chinese version with meanings equivalent to the original.

The backward translation from the first Chinese version to English was implemented by a pediatrician who had no access to the original US English version of the questionnaire and was bilingual in English and Chinese. The backward translated version was compared with the original source version by the multidisciplinary team. Any misunderstandings, mistranslations or inaccuracies were rectified after discussion and asking for the authorship’s guidance by e-mail. The second Chinese version was then produced.

During forward and backward translations, we made several modifications. Firstly, we changed all the wh-questions into general questions or declarative sentences. Additionally, based on the opinion of the original author of the scale, Professor Varni, we gave an explanation behind the item “The preparation provided for you about what to expect during tests and procedures” of “Communication” subscale, stating that “how well did the medical staff explain to the parents on what would happen to their child during the medical test and medical procedures”.

The preliminary test was conducted on 32 parents of pediatric patients with chronic diseases in order to obtain the comprehensibility of each item and the acceptability of the questionnaire. The second Chinese version of the scale was suitably revised according to the result of preliminary test. The average completion time of the second Chinese version of the scale was 15.20 minutes. There was no missing value in the questionnaire except the two items: “The sensitivity shown to you and your family during your child’s treatment” in “Inclusion of family” subscale, and “How much time the staff took to help you with your child coming back home” in “Technical skills” subscale. The two items were classified as difficult to understand since they were literal translated into Chinese. Based on the meaning of the questions and the opinion of the original author of the scale, we revised the two items as: “The sensitivity of the staff that showing loving care for you and your family during your child’s treatment” and “How much time the staff took to make your child improved or well-healed”. The final Chinese version was produced and to be field-tested in the current study.

#### Data analysis

The demographic characteristics of the parents and children were reported by descriptive analysis. Score distribution was evaluated by assessing the presence of floor and ceiling effects (>25% of the respondents have the minimum and/or maximum score) [23]. Continuous numerical variables were displayed as median, upper quartile and lower quartile as they did not obey normal distributions (the skewness values ranged from −0.840 to −0.206) while categorical variables were displayed as observed frequencies and proportions [18].

We assessed feasibility, reliability and validity of the PedsQL™ 3.0 Healthcare Satisfaction Generic Module. Feasibility was determined from the response rate, average completion time of the questionnaires and the percentage of missing/*not applicable* values for each item.

Scale internal consistency reliability was assessed by calculating Cronbach’s alpha coefficient and an alpha greater than or equal to 0.70 was considered acceptable [24]. Test-retest reliability of the scale was evaluated with the Intra-class Correlation Coefficient (*ICC*) and the value greater than or equal to 0.80 was considered adequate [25].

Content validity was assessed by item-subscale correlations (Spearman’s rank correlations). There was good scaling success when items had stronger correlations with their hypothesized subscales than those with other subscales.

Confirmatory Factor Analysis (CFA) was performed to assess construct validity of the scale [26]. CFA is a means of testing the hypothesis that the observed variables (items) are indirect measures of hypothesized latent variables (subscales) [27]. The CFA model was fit to the data using maximum likelihood (ML) estimation [27]. Goodness of fit was evaluated using the indexes including Chi-square (***χ***^***2***^), ***χ***^***2***^/***df*** ratio, Root Mean Square Error of Approximation (*RMSEA*), Non-Normed Fit Index (*NNFI*), Comparative Fit Index (*CFI*), Adjusted Goodness of Fit Index (*AGFI*) and Standardized Root Mean Square Residual (*SRMR*) [19,28]. A ***χ***^***2***^/***df*** ratio value of 5.00 or lower demonstrated an acceptable model fit [29]. The values of *RMSEA*, *NNFI*, *CFI*, *AGFI* and *SRMR* were in the range of 0 to1. For *NNFI* and *CFI*, a value of 0.90 or greater indicated good model fit [30]. A *RMSEA* value below 0.08 and an *AGFI* value above 0.85 were commonly considered adequate model fit [26]. A good model fit between the target model and the observed data is distinguished by *SRMR* values below 0.08 [30]. All of the goodness of fit indexes mentioned above were used in this study.

Analyses were performed using SPSS 18.0 and LISREL 8.8 for Windows.

## Results

### Subjects and feasibility

The survey took place from April 2012 to July 2012. Table 1 lists the descriptive analysis of the whole sample’s demographic characteristics. The majority of the subjects were mothers. On the item of “Family economic condition”, more than 90% of the subjects chose “intermediate to poor.” Among the children, More than 65% patients were male. The average age of the pediatric patients was 6.42 years old (SD = 3.93) and over 60% of them were younger than 7 years old.

**Table 1:** Demographic characteristics of the sample

**Demographic Characteristics**	***N***	**%**
**Characteristics of Parents**		
**Relationship to Patient**		
Mother	219	61.9
Father	118	33.3
Grandfather	3	0.8
Grandmother	4	1.1
Others	5	1.4
Not reported	5	1.4
**Education Level of the Respondents**		
Primary school degree or below	38	10.7
Junior high school	120	33.9
Senior high school or technical secondary school	85	24.0
Junior college	53	15.0
Bachelor degree or above	30	8.5
Not reported	28	7.9
**Family Economic Condition**		
Rich	3	0.8
Above the average	10	2.8
Intermediate	110	31.1
Under the average	105	29.7
Poor	105	29.7
Not reported	21	5.9
**Characteristics of Children**		
**Gender**		
Male	233	65.8
Female	121	34.2
**Ages (years)**		
2 ~ 4	147	41.5
5 ~ 7	86	24.3
8 ~ 12	86	24.3
13 ~ 18	35	9.9
**Diseases**		
Hematologic diseases/Tumor	226	63.8
Cardiovascular diseases	78	22.0
Nephritic syndrome	39	11.0
Asthma	6	1.7
Diabetes	5	1.4
**Hospitalization Duration (days)**		
3 ~ 30	166	46.9
31 ~ 90	79	22.3
91 ~ 180	55	15.5
>180	24	6.8
Not reported	30	8.5

The response rate was 94.4%. There were 375 parents of children with chronic diseases participated in the study. 354 subjects completed the questionnaire, 18 parents declined to participate, and 3 participants answered less than 50% of the items. Since a *not applicable* option was added for subjects if the item was not suitable for them, there was no missing value. Most of the items had few *not applicable* values except the items in Emotional Needs subscale. The *not applicable* rate of each item ranged from 0.0% to 24.3% (Table 2). Three of the items had high *not applicable* rates (more than 10%): “The amount of time given to your child to play, talk about her/his feelings, and any questions she/he may have”, “The amount of time spent helping your child with going back to school” and “The amount of time spent attending to your child’s emotional needs”. The average completion time of the questionnaire was 14.17 minutes (SD = 7.06).

**Table 2:** **Item-subscale correlations of the PedsQL**^**TM**^**3.0 healthcare satisfaction generic module**

**Items**	**Information**	**Inclusion of Family**	**Communication**	**Technical Skill**	**Emotional Needs**	**Total Satisfaction**	N/A*(%)
**Information**							
1.information provided about diagnosis	**0.90**	0.76	0.73	0.73	0.72	0.72	0.3
2.information about treatment and course	**0.91**	0.79	0.76	0.74	0.74	0.75	0.8
3.information about side effects of the treatment	**0.89**	0.74	0.73	0.70	0.69	0.66	0.8
4.how soon information was given about test results	**0.88**	0.73	0.69	0.67	0.70	0.68	1.4
5.how often are updated about your child’s health	**0.92**	0.80	0.77	0.76	0.74	0.75	2.0
**Inclusion of Family**							
6.sensitivity shown to you and your family	0.73	**0.88**	0.75	0.72	0.70	0.75	0.0
7.willingness to answer your questions	0.77	**0.92**	0.74	0.69	0.67	0.73	0.6
8.include your family in discussion	0.81	**0.92**	0.78	0.79	0.77	0.77	0.8
9.time given you to ask questions	0.78	**0.91**	0.80	0.74	0.71	0.73	1.1
**Communication**							
10.explain health condition and treatment to child	0.71	0.75	**0.85**	0.79	0.71	0.71	8.8
11.explain health condition and treatment to you	0.72	0.75	**0.85**	0.74	0.69	0.72	0.6
12.listen to you and your concerns	0.70	0.76	**0.87**	0.72	0.72	0.69	1.4
13 preparation provided for you about what to expect during tests and procedures	0.62	0.64	**0.83**	0.63	0.65	0.64	0.3
14 preparation provided for child about what to expect during tests and procedures	0.73	0.71	**0.87**	0.69	0.70	0.68	7.3
**Technical Skill**							
15.respond to child’s needs	0.75	0.75	0.79	**0.93**	0.76	0.74	2.3
16.efforts to keep your child comfortable	0.73	0.73	0.77	**0.93**	0.77	0.72	1.4
17.time to help your child back home	0.76	0.77	0.76	**0.92**	0.74	0.77	3.7
**Emotional Needs**							
18.time given to your child to play, talk about her/his feelings, and any questions she/he may have	0.72	0.73	0.74	0.72	**0.90**	0.68	10.7
19.time spent helping your child back to school	0.74	0.73	0.76	0.74	**0.92**	0.71	24.3
20.time spent attending to child’s emotion needs	0.76	0.73	0.77	0.79	**0.95**	0.73	11.9
21.time spent attending your emotion needs	0.75	0.76	0.76	0.79	**0.96**	0.74	9.6
**Total Satisfaction**							
22.the overall care your child is receiving	0.71	0.73	0.74	0.74	0.70	**0.92**	0.3
23.how friendly and helpful the staff is	0.70	0.74	0.73	0.72	0.69	**0.89**	0.3
24.the way your child is treated at the hospital	0.72	0.74	0.70	0.71	0.70	**0.90**	0.6

Values denote Spearman’s rank correlation coefficients (*P* < 0.01).

Bolded values represent correlations between items and their hypothesized subscales.

*N/A = *Not Applicable.*

### Descriptive analysis

Table 3 presents the median, upper and lower quartiles, and the floor and ceiling effects on each subscale score and total score of the Healthcare Satisfaction Generic Module.

**Table 3:** Subscales descriptions and reliability for the PedsQL™ 3.0 healthcare satisfaction generic module

**Subscale**	***N***	***Median (Q***_***L***_***,Q***_***U***_***)***	***% Floor***	***%Ceiling***	***Cronbach’s α***	***ICC (95% CI)***
Total Score	354	75.00(69.79,87.64)	0.0	9.6	0.94	0.92(0.86,0.96)
Information	354	75.00(70.00,85.00)	0.6	16.9	0.93	0.87(0.75,0.93)
Inclusion of Family	354	75.00(68.75,93.75)	0.3	22.9	0.91	0.81(0.65,0.90)
Communication	350	75.00(70.00,85.00)	0.0	17.2	0.92	0.87(0.75,0.93)
Technical Skills	351	75.00(75.00,91.67)	0.6	21.2	0.95	0.90(0.80,0.95)
Emotional Needs	321	75.00(62.50,81.25)	0.3	15.8	0.89	0.86(0.74,0.93)
Overall Satisfaction	353	75.00(75.00,91.67)	0.0	24.6	0.98	0.90(0.82,0.95)

*Q*_*L*_ = lower quartile, *Q*_*U*_ = upper quartile.

*%Floor, %Ceiling* = percentage of scores at the extremes of the scaling range.

*ICC* = Intra-class Correlation Coefficient, *CI* = Confidence Interval.

### Reliability

Cronbach’s alpha coefficients are presented in Table 3, which were used to evaluate the internal consistency reliability of the PedsQL™3.0 Healthcare Satisfaction Generic Module. The Cronbach’s alpha coefficients of all subscale scores and total score exceeded 0.70. The test-retest reliability was examined by calculating *ICC*s of all subscales, which were all higher than the minimum recommended standard of 0.80 (Table 3).

### Item-subscale correlations

Spearman’s rank correlation coefficients between items and subscale scores are showed in Table 2. There were higher correlation coefficients between items and their hypothesized subscales than those with other subscales.

### Construct validity

CFA was performed to assess construct validity of the scale. A six-factor model was established based on the original scaling structure and the Goodness-of-Fit results were presented in Table 4. The *CFI* value, and *NNFI* value were greater than 0.90, *RMSEA* and *SRMR* was less than 0.08, while *AGFI* did not reach the minimum standard of 0.85.

**Table 4:** Goodness-of-Fit for the six-factor model

***χ***^***2***^	***df***	***χ***^***2***^***/df***	***RMSEA (95% CI)***	***CFI***	***NNFI***	***AGFI***	***SRMR***
765.49	237	3.23	0.078 (0.072,0.084)	0.99	0.99	0.81	0.027

*χ2* = Minimum Fit Function Chi-square; *df* = degree of freedom;

*RMSEA* = Root Mean Square Error of Approximation; *CI* = Confidence Interval;

*CFI* = Comparative Fit Index; *NNFI* = Non-Normed Fit Index;

*AGFI* = Adjusted Goodness of Fit Index; *SRMR* = Standardized Root Mean Square Residual.

## Discussion

The PedsQL™ 3.0 Healthcare Satisfaction Generic Module, a module of the PedsQL™ Measurement Model, is an instrument to measure the parents’ satisfaction with the healthcare for their children with chronic diseases and has been adapted for use in other countries [31]. This study aimed to develop a Chinese version of the PedsQL™ 3.0 Healthcare Satisfaction Generic Module and evaluate the psychometric properties of the scale. To our knowledge, this is the first report of psychometric properties of the PedsQL™ 3.0 Healthcare Satisfaction Generic Module in a pediatric chronic diseases sample in China.

In the standard procedure of cross-culture adaptation, we strictly followed the PedsQL™ Measurement Model Translation to finalize the Chinese version. The result showed that the scale was a feasible and practical instrument with high response rate and short completing time. Most of the items had few *not applicable* values except the items in the Emotional Needs subscale, such as “The amount of time given to your child to play, talk about her/his feelings, and any questions she/he may have”, “The amount of time spent helping your child with going back to school” and “The amount of time spent attending to your child’s emotional needs.” The higher percentage of *not applicable* values was found to be primarily from the lower age groups, with parents reasoning that their children were too young to go to school. Young pediatric patients also had difficulty talking about their feelings or emotional needs. In addition,some parents found the item “The amount of time spent helping your child with going back to school” hard to understand. This finding was consistent with the results seen in the Brazilian version of the scale [31]. The results indicated that some suitable modifications were necessary for the items of Emotional Needs subscale. Future studies may develop different age-group scales according to their own characteristics.

Additionally, the study results showed that the Chinese version of PedsQL™ 3.0 Healthcare Satisfaction Generic Module was a reliable and valid instrument to assess the parents’ satisfaction with the healthcare for their children with chronic diseases. The internal consistency reliability of the scale was evaluated by Cronbach’s alpha coefficients. All Cronbach’s alpha coefficients exceeded the recommended standard of 0.70 in all subscales, indicating adequate reliability of the Chinese version scale. This finding was roughly consistent with the results reported by a prior study [31]. Test-retest reliability was examined using *ICC*s. All *ICC*s of the subscales were higher than 0.80, which demonstrated a good test-retest reliability of the scale.

The results of Spearman’s rank correlation coefficients between items and subscale scores indicated good scaling success since items had high correlations with their hypothesized subscales, which were stronger than those with other subscales.

The CFA was performed and the results indicated that the model fit the data well according to all the Goodness of Fit Statistics. The *RMSEA*, *CFI* and *SRMR* all reached the recommended standards. The adequate construct validity confirmed the premeditated hypothesis of the Chinese version scale.

Our study has several potential limitations. Firstly, this study was conducted only in the highest level hospitals and the results may not be generalized to other level hospitals in China. We recommend future studies to evaluate the psychometric properties of the Chinese version scale in the samples of other level hospitals in China. Secondly, convenience sampling was used in this study, and there may be sampling bias. Thirdly, the variety of chronic diseases was limited in the present study. Parents of pediatric patients with other chronic diseases should be recruited in future studies. Fourthly, only 35 children were 13–18 years old, therefore, the results of this study may have little generalizability for those high school students. Fifthly, the responsibility to change (sensitivity) of the scale was not evaluated. Intervention for the healthcare service can be implemented to assess the responsibility to change (sensitivity) of the Chinese version scale in future studies. Moreover, the parental satisfaction was assessed to reflect the healthcare satisfaction, but to some extent, it may be different from the patient’s satisfaction. In order to evaluate the healthcare satisfaction comprehensively, the scale of pediatric patient’s satisfaction (especially the older pediatric patient’s satisfaction) could be developed in the future studies.

## Conclusions

As the first study to evaluate the psychometric properties of the Chinese version PedsQL™ 3.0 Healthcare Satisfaction Generic Module, our results demonstrated that the Chinese version scale is a promising instrument with adequate reliability and validity to measure the parents’ satisfaction with the healthcare for their children with chronic diseases. Future studies should focus on evaluating the responsibility to change (sensitivity) of the scale and testing on parents of pediatric patients with other chronic diseases and in a variety of different level hospitals in China.

## Abbreviations

PedsQL™: Pediatric quality of life inventory™; ICC: Intra-class correlation coefficient; CFA: Confirmatory factor analysis; ML: Maximum likelihood; RMSEA: Root mean square error of approximation; NNFI: Non-normed fit index; CFI: Comparative fit index; AGFI: Adjusted goodness of fit index; SRMR: Standardized root mean square residual.

## Competing interests

The authors declare that they have no competing interests.

## Authors’ contributions

JL conceptualized and designed the study, acquired, analyzed and interpreted the data, and drafted the manuscript. LY conceptualized and designed the study, acquired the data and revised the manuscript. YW and YL acquired data and revised the manuscript. YH conceptualized and designed the study, supervised the data analysis and revised the manuscript. All authors read and approved the final manuscript.
